# Supplementary material for: Cohesin depleted cells rebuild functional nuclear compartments after endomitosis
Source: Nat Commun. 2020 Dec 1;11:6146. doi: 10.1038/s41467-020-19876-6 (PMC7708632; doi:10.1038/s41467-020-19876-6)
Supplement: Supplementary file 1 — Supplementary Information [file 41467_2020_19876_MOESM1_ESM.pdf]

Supplementary information

Cohesin depleted cells rebuild functional nuclear compartments after endomitosis

Cremer et al.

Supplementary Figures1-8
Supplementary Notes 1-5
Supplementary Tables1-2
Supplementary References

Supplementary figures with explanations

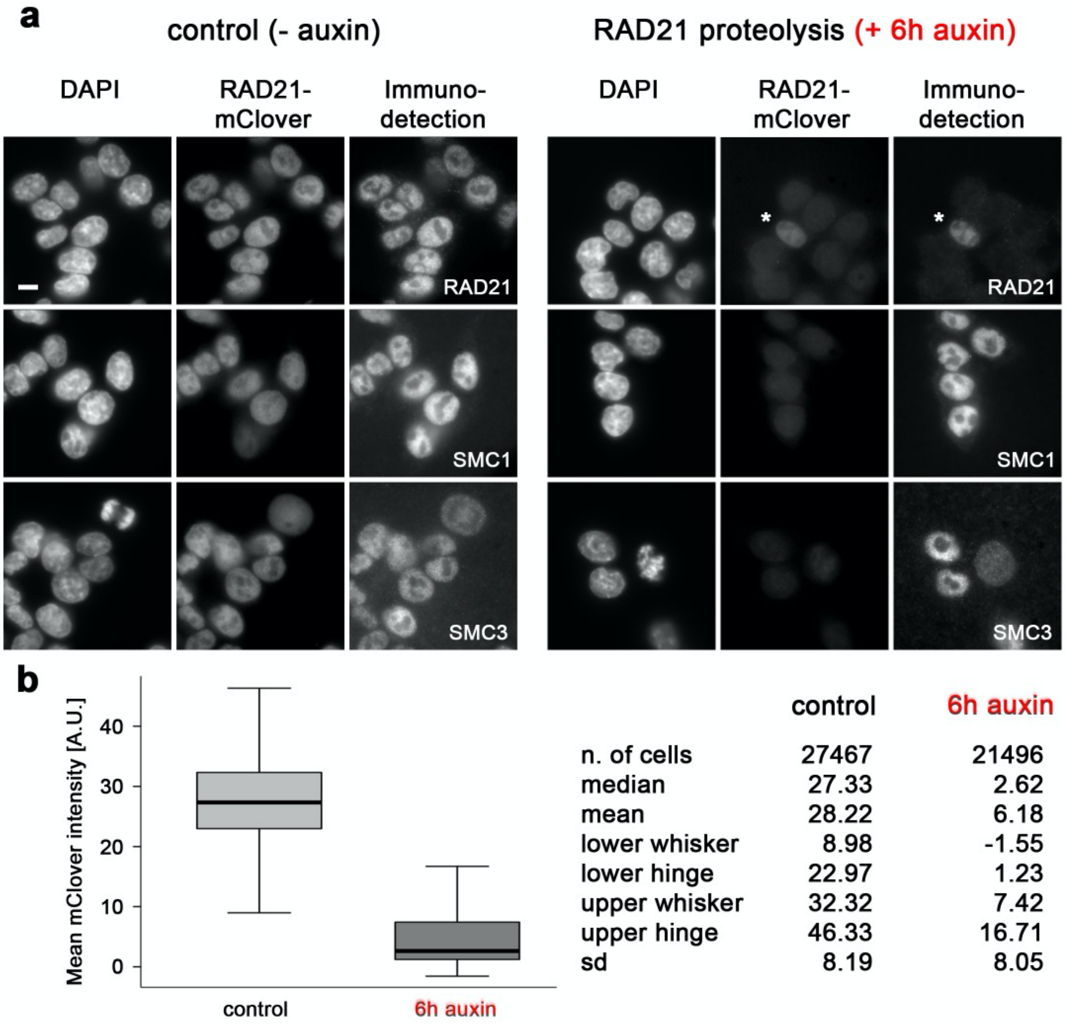

	control	6h auxin
n. of cells	27467	21496
median	27.33	2.62
mean	28.22	6.18
lower whisker	8.98	-1.55
lower hinge	22.97	1.23
upper whisker	32.32	7.42
upper hinge	46.33	16.71
sd	8.19	8.05

Supplementary Fig. 1: RAD21-mClover proteolysis under auxin treatment

(a) Immunodetection of the three major cohesin subunits RAD21, SMC1 and SMC3. Cells treated 6h with auxin (right panel) confirm the loss of RAD21 immunostaining in accordance with loss of RAD21-mClover fluorescence. Asterisk indicates a cell escaping RAD21 proteolysis. SMC1 and SMC3 immunostaining is maintained. Images are representative for two independent experiments. Scale bar: 5 µm. **(b)** Averaged RAD21-mClover intensities recorded by high-throughput imaging from single cells of untreated controls (median = 27.33 A.U., n=27467 cells) and auxin treated cells fixed after 6h in 500 μM auxin (median = 2.62 A.U., n=21496 cells). Significance was tested by a two-sided Wilcoxon test ($p < 0.0001$) without correction for multiple testing. For details, see Methods. The small overlap between cohorts is likely due to the small fraction of cells escaping AID induced RAD21 proteolysis in the auxin cohort and to cells that lack RAD21-mClover expression in control cells (compare Supplementary Fig. 2). Data in b are represented as boxplot where the middle line indicates the median, the lower and upper hinges correspond to the 25% and 75% quartiles, the upper whisker extends to the largest value no further than 1.5 x IQR (inter-quartile range) from the hinge and the lower whisker extends to the smallest value from the hinge at most 1.5 x IQR. Source data are provided as a Source Data file

.

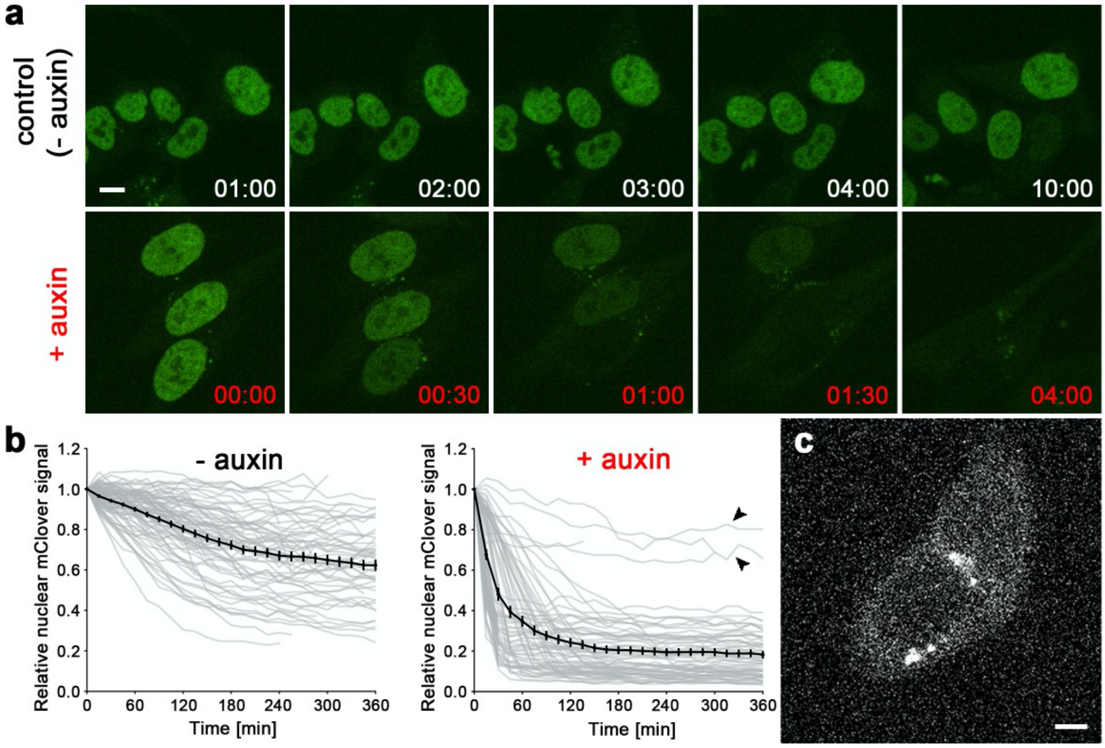

Supplementary Fig. 2: Time course and quantitative measurement of auxin induced RAD21-mClover degradation based on single cell analyses from live cell observations

(a) Upper row: selected time points of time lapse imaging (Δt=15min) in control cells (- auxin) show largely persistence of RAD21-mClover fluorescence over 10 h. Lower row: gradual decrease of RAD21-mClover fluorescence shown for selected time points in auxin treated cells recorded under same imaging conditions. RAD21-mClover fluorescence appears accomplished between time point 01:30-04:00 after addition of 500 µM auxin. Scale bar: 5 µm. **(b)** Quantitative analysis of single cell nuclear RAD21-mClover fluorescence recorded over 6h from live cell experiment shown in (a). Time course of nuclear fluorescence was analyzed by use of automated image data analysis and segmentation tools (see materials and methods). In control cells nuclear fluorescence decreases to a mean value of ~60% of the starting value due to imaging related bleaching. Auxin treatment reduces RAD21-mClover fluorescence to an average of ~20% of the starting value, this value settles after ~3h. Arrows indicate cells that apparently escaped auxin degradation in this experiment. Control cells: n=82; auxin-treated cells: n=69. Data are represented as mean ± SEM. **(c)** Fluorescent degradation products of RAD21-mClover around the nucleus (cf. bright speckles) and in the cytoplasm, that can affect the results of the automated analysis. Data in a-c show representative cells from one of the three independent experiments. Scale bars: 5 µm. Source data are provided as a Source Data file.

Supplementary Note 1: AID triggers proteasomal degradation of a tagged protein by ubiquitination, while its expression continues. It can be assumed that the remaining fluorescence in auxin treated cells mostly originates from RAD21-mClover in proteasomes associated with the cytoskeleton, centrosomes and the outer surface of the endoplasmic reticulum [1], while the fraction of RAD21 within an intact cohesin ring is neglectable. Note also the observation of a residual fluorescence of ~10% of mClover signals was described in the original publication by [2] after addition of 500 µM auxin.

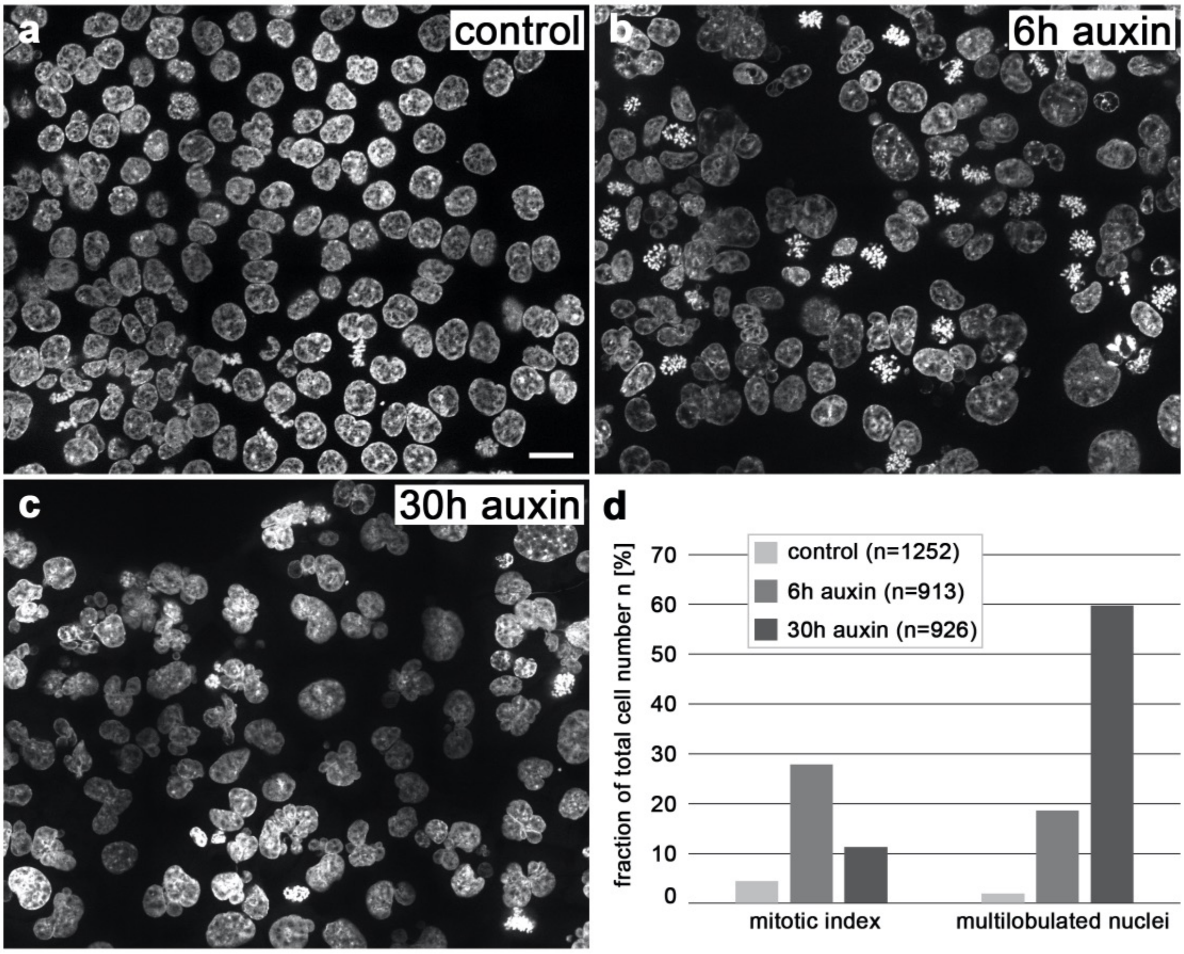

Supplementary Fig. 3: Transient increase of mitotic index and fraction of MLN after cohesin depletion

(a-c) Representative images (z-projections from two optical sections with Δz = 6 µm) of **(a)** DAPI stained control nuclei; **(b)** nuclei fixed 6h after auxin treatment showing the accumulation of mitoses; **(c)** nuclei fixed 30h after auxin treatment with highly enriched MLN. Scale bar: 20 µm. Images are representative images from two independent experiments. **(d)** Quantification of mitotic index and fraction of MLN in control and cohesin depleted nuclei (control: light gray, n=1252, 6h auxin: gray, n=913, 30h auxin: dark gray, n=913 cells). Data are represented as mean. Apoptotic and morphologically inconspicuous nuclei with distinct micronuclei were excluded from the MLN fraction. Source data are provided as a Source Data file.

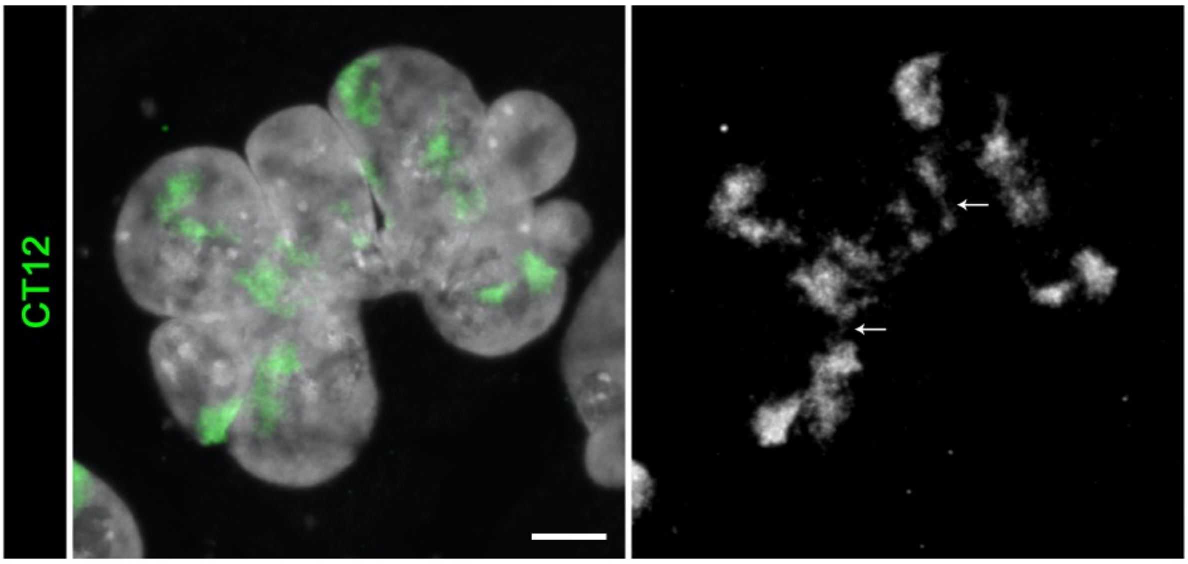

Supplementary Fig. 4: Selective presentation of chromosome territory (CT) 12 reveals a tearing apart of painted regions

(Left) Exemplary cohesin depleted postmitotic MLN (DAPI: gray, shown also in Fig. 2d) with apparently >4 variably sized painted regions for CT 12 in different lobuli (green). (Right) Selective presentation of painted regions (gray) reveal thin chromatin bridges between them (arrows). Scale bar: 5 μm

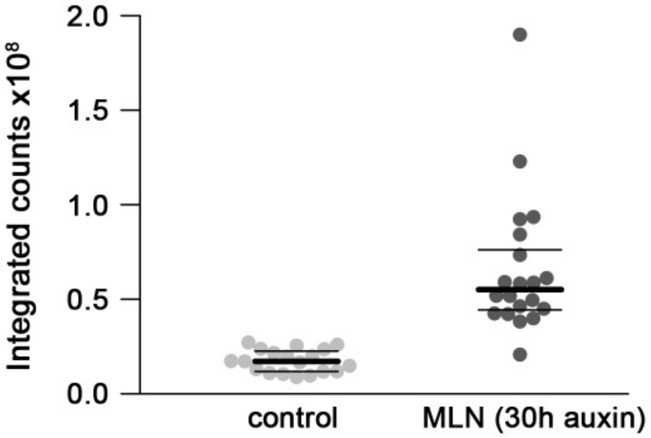

Supplementary Fig. 5: DNA content measurements in controls and cohesin depleted postmitotic multilobulated nuclei (MLN)

DNA content of single interphase nuclei based on integrated DAPI intensities of confocal sections in control cells (n=19, light gray) and postmitotic cohesin depleted MLN after 30h auxin treatment (n=20, dark gray). Control cells show a narrow distribution reflecting the DNA content in G1, S, G2 phase (1n – 2n). MLN show an overall increased DNA content with a wide range. Note that these cells arise from an endomitosis with a 2n DNA content. MLN can pass through another full round of replication (compare Fig. 6E in the main text) increasing their DNA content up to 4n. Data are represented as single data points with the median as thick line and the first and third quartiles as thin lines. Source data are provided as a Source Data file.

Supplementary Note 2: DAPI based single cell DNA content measurements may not reflect the absolute DNA content and a quantitative comparison of cells with highly different morphologies should be interpreted with caution [4]. However, the increased DNA content of cohesin depleted MLN is robust.

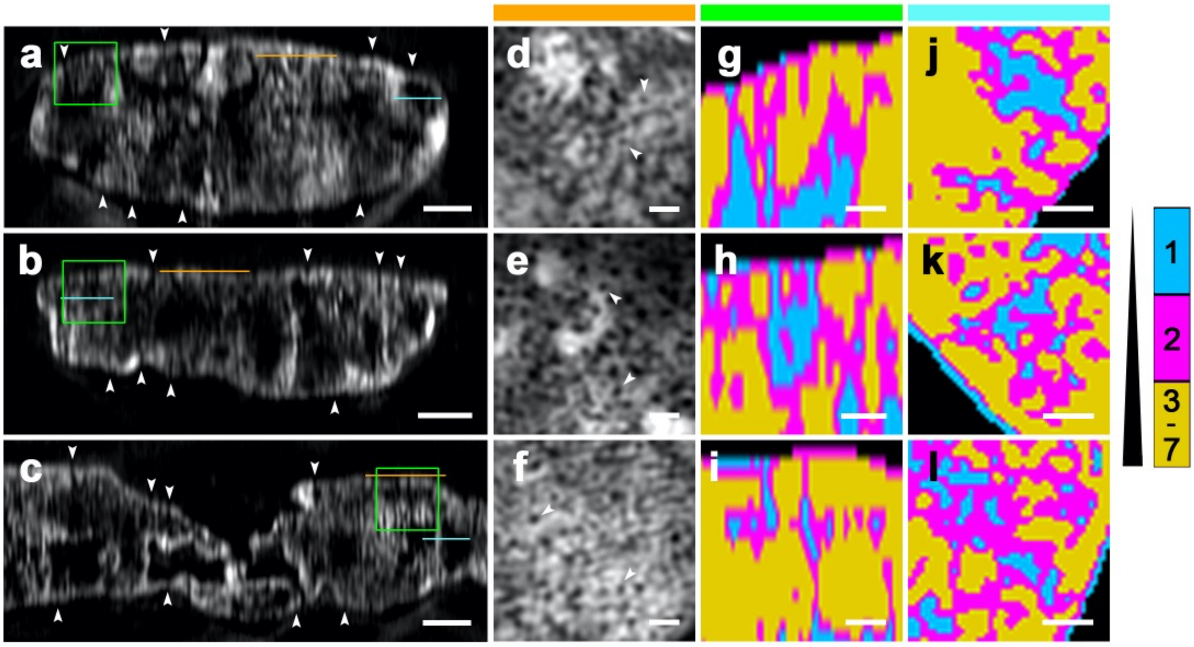

Supplementary Fig. 6: Maintenance of a 3D network of the interchromatin compartment (IC) channel system after cohesin depletion and its reconstitution in MLN after mitosis

(a-c) Z-sections from DAPI stained nuclei shown in Fig. 3a-c demonstrate IC-channels (arrowheads) extending between nuclear envelope associated chromatin domains into the nuclear interior, where they form wide IC-lacunas. Scale bars: 2 µm. **(d-f)** Apical XY-sections from respective nuclei (indicated as orange lines in a-c) delineating the passage of IC channels through peripheral heterochromatin at the nuclear lamina (not shown) where IC-channels are noted as black holes. Scale bars: 0.5 µm. **(g-i)** Inset magnifications from z-sections of nuclei shown in a-c (indicated as green frames) presented with three color coded DAPI intensity classes 1 (blue), 2 (purple), 3-7 (merged in beige; for description see Fig 3) further illustrate the extension of IC-channels from the nuclear periphery into the interior and the expansion of the IC into extended IC-lacunas (class 1) both within the control nucleus **(g),** and cohesin depleted pre- and postmitotic nuclei **(h,i).** The apparent predominance of a vertical channel alignment in z-sections is a consequence of the lower resolution in z (axial, ~250 nm) compared to xy (perpendicular to the optical axis, ~125 nm) [5]. **(i-l)** XY-sections from respective nuclei (indicated as blue lines in a-c) demonstrate the occurrence of transversal channels from the lateral periphery towards the nuclear interior in line with a three-dimensional network. Scale bars: 0.5 µm.

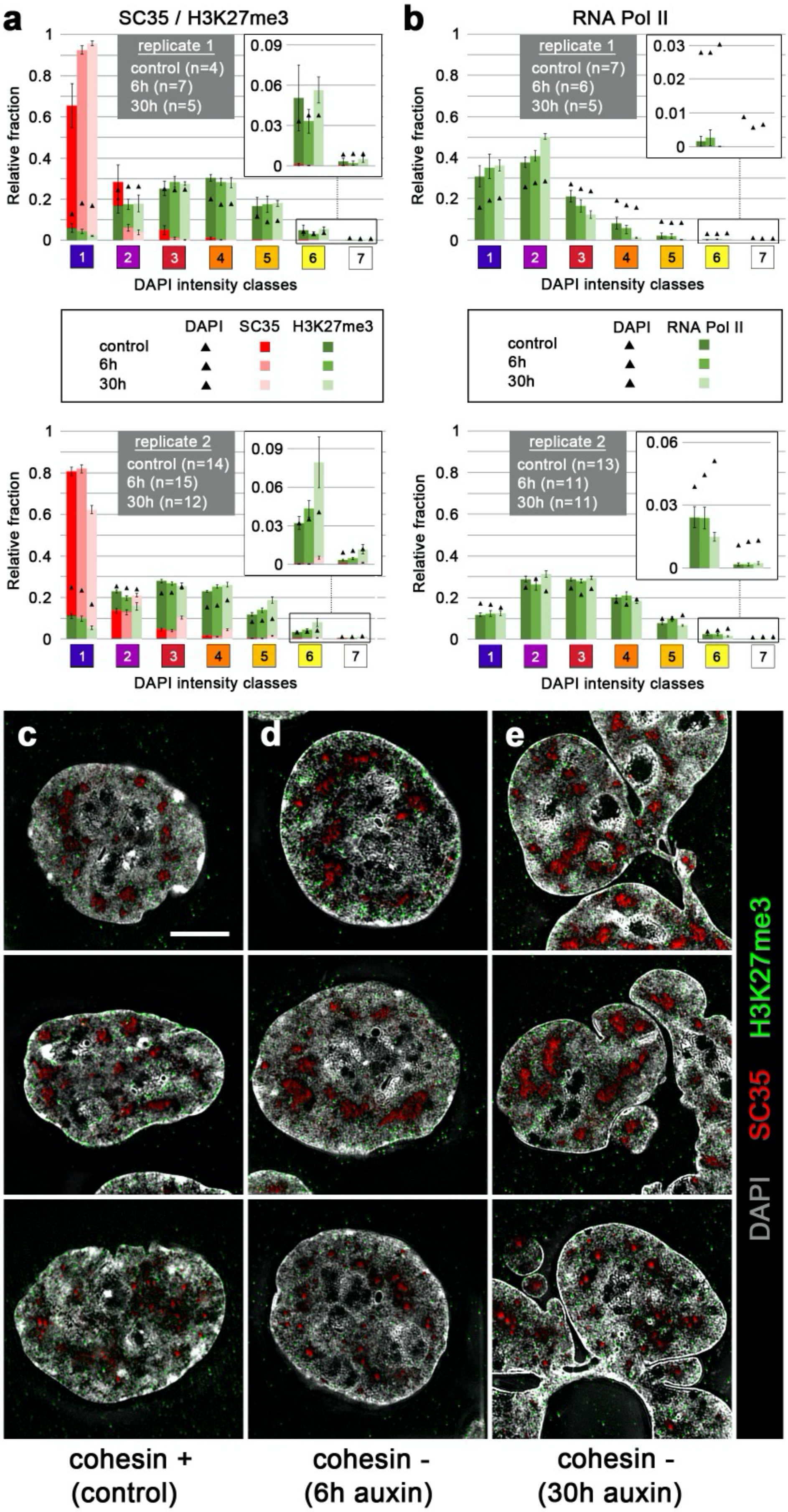

Supplementary Fig. 7: **Interexperimental variability of quantitative mapping of SC35, H3K27me3, RNA Pol II on chromatin compaction maps** (legend continued on next page)

This figure provides an extension of data shown in Fig. 4 to exemplify interexperimental variability. **(a,b)** Separate presentation of replicates 1 and 2 which were performed about one year apart from each other with relative enrichments and depletions of **(a)** SC35 (red) and H3K4me3 (green) (rep1: n=4, rep2: n=14; 6h: rep1: n=7, rep2: n=15; 30h: rep1: n=5, rep2: n=12 cells) and **(b)** RNA Pol II (green) (control: rep1: n=7, rep2: n=13; 6h: rep1: n=6, rep2: n=11; 30h: rep1: n=5, rep2: n=11 cells) in the 7 DAPI intensity classes marked as black triangles (for explanation of details see the main text). Data in (a) and (b) are represented as mean ± SEM. Source data are provided as a Source Data file. **(c-e)** Mid-SIM sections from **(c)** control nuclei**, (d)** cohesin depleted nuclei fixed after 6 h auxin treatment**, (e)** partial sections of post-endomitotic MLN fixed 30 h after auxin treatment are shown with DAPI stained DNA (gray), immunostained SC35 (red) and H3K27me3 (green). SC35 marked speckles in IC lacunas demonstrate compaction differences between speckles in both control and cohesin depleted nuclei.

Supplementary Note 3: Technical parameters for image recording and quantitative image analysis were kept constant in both experiments. Therefore, they are an unlikely source to explain interexperimental differences. Instead, unperceived biological differences between the cultures studied in the two experiments may explain why RNA Pol II was particularly enriched in classes 1 and 2 (IC and lining chromatin) in one experiment, and in classes 2 and 3 in another experiment. Although culture conditions appeared to be the same, parameters, which may affect the dynamics of higher order chromatin arrangements in cycling cells have remained elusive. The cultures were not synchronized and the two time points chosen for fixation of auxin treated cells (6h and 30h) have to be considered as snap-shots.

Different conformations of SC35 (shown in A-C) constituting a component in nuclear speckles composed of several protein complexes in a multilayered organization, was described [3], but underlying causes have remained elusive.

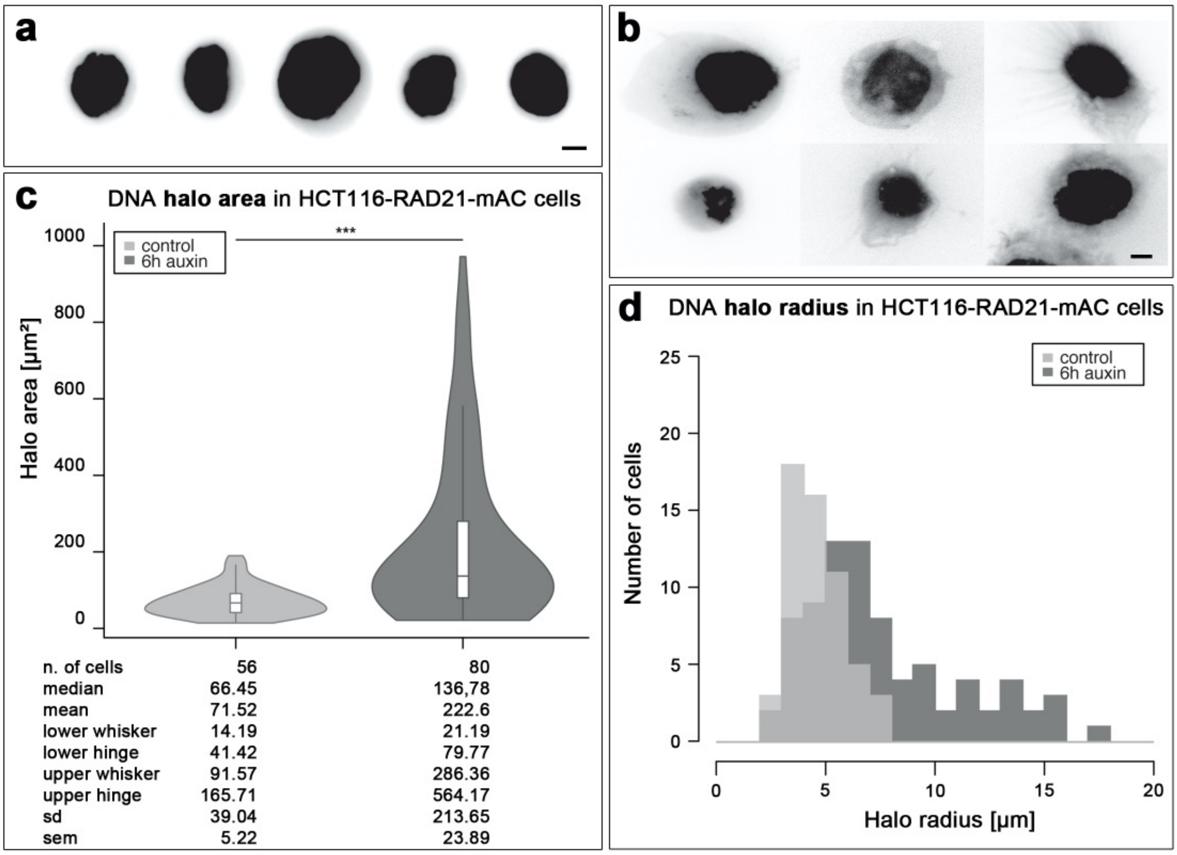

Supplementary Fig. 8: Enlargement of DNA Halos after cohesin depletion

(a-b) Representative images of DNA halos stained with DAPI recorded from four independent experiments. The faded DNA halo surrounding a brighter insoluble nuclear scaffold corresponds to the DNA loops, whose extent reflects the degree of structural organization of chromatin. **(a)** Typical nuclei from control cells with small, rather uniform, and well delimited halos. **(b)** Halos of cohesin depleted cells show variable shapes and size, often ending up in extruded bundles of DNA fibers. Scale bar: 5 µm. **(c)** Violin plot showing the differences in area of the DNA halo between control (light gray) and cohesin depleted cells (6h of auxin treatment, gray), determined as described in Methods. Cohesin depleted cells show up to five times larger halos. p-value < 0.0001 (***) using a two-sided Wilcoxon test without correction for multiple testing. Data in c are represented as violin plot where the middle line indicates the median, the lower and upper hinges correspond to the 25% and 75% quartiles, the upper whisker extends to the largest value no further than 1.5 x IQR (inter-quartile range) from the hinge and the lower whisker extends to the smallest value from the hinge at most 1.5 x IQR. The violin represents the data distribution. **(d)** Distribution of radial values of DNA halos for the two populations shown in (c). Source data are provided as a Source Data file.

Supplementary Table 1: Glossary with explanatory notes of current terminologies for higher order chromatin structures and nuclear compartmentalization

Supplementary Note 4: Microscopic and Hi-C studies have been used to explore nuclear landscapes in many species and cell types. These studies have yielded different terminologies, whose relationships are by no means obvious. This table serves as a glossary of terms currently used in (a) microscopic studies and (b) in Hi-C and related studies. The lack of a common terminology in 4D nucleome research, except for a general consent on chromosome territories (CTs), reflects unsolved gaps and inconsistencies regarding the 3D and 4D (space-time) organization of structural entities and their suspected functional roles.

(a) Terminology based on microscopic studies

**nucleosome clutches (NCs) nanodomains (NDs)** *6,7*	NCs/NDs with sizes of a few kb may represent the smallest structural entities of chromatin organization above the level of individual nucleosomes. Nucleosomes in NCs/NDs may be so densely packed that macromolecular aggregates can exert their functions only at the surface of NCs/NDs. 4D organization of NCs/NDs has remained elusive.
**chromatin loops** *8*	The 3D and 4D structure of chromatin loops is currently studied with super-resolved fluorescence microscopy and electron microscopy. The actual compaction of DNA/chromatin within loops is not known.
**~1 Mb chromatin domains (CDs)** *9-13*	The microscopic definition of ~1Mb chromatin domains (CDs) in early microscopic studies was based on replication domains (RDs) (see below). CDs with less compact chromatin are enriched with epigenetic marks of transcriptionally competent chromatin, whereas CDs with chromatin of higher compaction are enriched with marks for repressed chromatin. In a most recent study, Miron et al. described CDs as ~200-300 nm-wide mesoscale domains, composed of aggregated nucleosomes with nanoscale functional topography independent of cohesin.
**TAD-like domains** *14,15*	TAD-like domains were demonstrated at the single cell level by optical reconstruction of chromatin architecture (ORCA). This approach combines oligopaint-FISH with super-resolution fluorescence microscopy. In cohesin depleted cells, a more stochastic placement of borders between TAD-like domains was detected.
**replication foci, replication domains (RDs)** *12,16-18*	RDs with an estimated DNA content of ~1Mb were first detected in mammalian cells by pulse-labelling of replicating DNA during S-phase with halogenated nucleotides. At high resolution, RDs represent assemblies of several replicons with ~100–200 kb. RDs are stably maintained over subsequent cell cycles independent of their association with the transcription machinery. Chromatin assembled in early replicating RDs is enriched with active genes, whereas mid- and late-replicating RDs comprise mostly repressed chromatin.
**chromatin domain clusters (CDCs) and CD chains** *9,13,19*	CDCs comprise a peripheral layer of low compacted, transcriptionally competent CDs and an internal core of more compact, transcriptionally repressed CDs. CDs form interlinked chains surrounded by an RNA-populated interchromatin compartment (see below).
**chromosome territory (CT)** *20*	Evidence for a territorial arrangement of chromosomes (CTs) in cell nuclei was obtained in a wide range of animal and plant species by microscopic studies.
**interchromatin compartment (IC)** *9,21*	The IC refers to a contiguous 3D channel network, connected to nuclear pore complexes and expanding throughout the nucleus between CDs and CDCs. The finest ramifications of the IC have not yet been defined, but may extend between neighboring nucleosome clutches (NCs) or nanodomains (NDs). Wider IC-lacunas harbor large macromolecular complexes, e.g. splicing speckles. The IC may serve as a 'road' system for import of macromolecules, distribution to macromolecular complexes to sites of need, and mRNA export.

**perichromatin region (PR)** *9,10*	The PR comprises low compacted, transcriptionally competent chromatin and lines the borders of IC channels and lacunas. It is easily accessible for factors and factor complexes pervading the IC or released from nuclear bodies and splicing speckles. The PR is enriched in regulatory and coding sequences of active genes and represents the nuclear subcompartment, where most of the transcription, splicing of primary transcripts, as well as transcription of regulatory RNAs takes place.
**active nuclear compartment (ANC)** *9,21*	The ANC is formed by the IC together with the PR.
**inactive nuclear compartment (INC)** *9,21*	The INC comprises both CDs with repressed genes located in the interior of CDCs ('facultative' heterochromatin) and clusters of 'constitutive' heterochromatin. Like the ANC, the INC is pervaded by IC-channels.

(b) Terminology based on Hi-C and biochemical evidence

**contact domains and boundaries** *22-24*	Contact domains refer to an interval exhibiting increased contact frequency between loci inside this interval versus outside, i.e an on-diagonal square in a Hi-C map. Contact domains detected in population Hi-C studies do not represent an individual chromatin structure, but rather a statistical feature of a cell population. Contact domains can form as a result of loop extrusion [22,24] or compartmentalization [23]. Boundaries detected in Hi-C experiments represent transition points between contact domains.
**loop domains** *22,24-26*	Loop domains refer to a subset of contact domains that exhibit additional increased frequency between the ends of the interval, i.e. peaks in the corner. They are frequently, but not exclusively anchored by a cohesin ring at a pair of convergent CTCF binding sites, and they are thought to form as a result of loop extrusion.
**compartment domains** *27*	Compartment domains are a subset of contact domains which, in addition to exhibiting increased contact frequency inside the domain interval versus outside, also exhibit an elevated contact frequency with other compartment domains of the same "type" as compared with other compartment domains of a different "type". This manifests as plaid/checkerboard patterns in a Hi-C map. Compartment domains can vary drastically in size, and compartment domains as small as with a DNA content of only ~10-15 kb have been detected in Drosophila with high-resolution Hi-C. Compartment domains form independently of cohesin-dependent loop extrusion, thus compartment domains are neither mutually exclusive nor hierarchical to loop domains, compartment domains can span multiple loop domains, multiple compartment domains of different types (for instance, active and repressed) can be spanned by a single loop domain, or a compartment domain and loop domain can be coincident.
**topologically associating domains (TADs)** *23,28-31*	TADs generally refer to intervals exhibiting increased contact frequency between loci inside the interval versus outside along with additional constraints that have varied depending on the study (for instance: some have indicated a general size range of few hundred kb to 1Mb; some have imposed the requirement that TADs be contained within a contiguous compartment interval of the same type; some have defined TADs as only those intervals that demonstrate increased within-interval contact frequency as a result of loop extrusion).

**replication domains (RD)** [32-38]	400-800kb sized RDs were first noted in repli-seq experiments as developmental switches - that is the unit of DNA that changes replication timing coordinately. The regulatory mechanism(s) that specify the replication timing of RDs in a timely manner despite stochastic origin firing are currently not known.
**compartment A**<br>**compartment B** [23,25,27,29,39,40]	Compartment A is globally defined by all compartment domains of subtype A, generally corresponding active chromatin, and compartment B by all compartment domains of subtype B, generally corresponding to repressed chromatin. Compartment A and B correspond to the two main pattern types observed in the plaid/checkerboard patterns in Hi-C maps. It has also been demonstrated that these two main compartment types can be further subdivided into subcompartments that correlate with distinct patterns of chromatin modifications on the basis of variations in contact patterns.
**chromosome territories (CTs)** [23]	CTs were demonstrated by enhanced contact frequency between all pairs of loci within a single chromosome in comparison with neighboring chromosomes.

Supplementary Note 5: A comparison of the terminologies described above demonstrates the strength and weaknesses of microscopic and Hi-C strategies. Whereas ensemble Hi-C has the tremendous advantage of revealing 3D DNA-DNA contact frequencies in a genome wide manner, advanced microscopy has started to pave the way to high-resolution studies of chromatin dynamics at the single cell level [13,41] and has become the method of choice to identify the 3D network of channels that connect higher order chromatin structures in the nuclear interior with the nuclear pores [21].
A common terminology should be based on clearly defined experimental approaches. For instance, a contact domain as defined in the glossary, does not depend on size scale, so no false sense of the size of the feature is implied that may be due just to the limitations of the resolution of the Hi-C map. In this sense, all loop domains, TADs and compartment domains can be subsumed under the heading of contact domains, notwithstanding major differences in their size ranges. Furthermore, the definition of a loop domain as a special sort of contact domain is independent of the question, whether a given loop is formed by CTCF/cohesin or other mechanisms. The 4D organization of such loops in living cells has remained elusive. Current perspectives range from an open architecture, where DNA targets in the loop interior are easily accessible for macromolecular complexes to highly compact structures, which constrains the accessibility of individual macromolecules and excludes larger macromolecular complexes. The history of the term chromosome can serve as a case in point for the importance to avoid an overloading of terms with unproven functional speculations. When Wilhelm Waldeyer (1836-1921) introduced this term in 1888 [42] he was aware of August Weismann's (1834-1914) ingenious, but highly speculative theory of heredity (reviewed in [43]). He even referred to Johann Friedrich Miescher's (1844-1895) discovery of "nuclein" and to Albrecht Kossel's (1853-1927) early publications on "histon" and "adenin" (reviewed in [44]). Waldeyer, however, preferred to propose the name chromosomes to emphasize the possibility of coloring the worm-like entities, seen during mitosis, by certain stains. This term has remained open for all conceptual changes, which happened thereafter with regard to the structure and function of chromosomes to the present day.

(c) Our preliminary attempt to integrate the different perspectives of microscopy and Hi-C and tentatively suggest the following hypotheses:

- Active and repressed CDs are located within the ANC and INC, respectively. We equate the ANC with compartment A and the INC with compartment B defined by Hi-C experiments.
- CDs within the ANC may be equated with compartment domains A, CDs located in the INC with compartment domains B.
- Based on evidence that RDs, CDs and compartment domains, but not TADs, were detected in cohesin depleted cells, we suggest to equate RDs with CDs and compartment domains rather than with TADs. This view is, however, challenged by the observation of TAD-like domains in experiments that combined oligo-paint FISH of sequence-defined TADs with super-resolved fluorescence microscopy. These experiments may indicate that the failure to detect TADs in cohesin-depleted cells in ensemble Hi-C experiments is not due to a real loss of structural entities, but to apparently random cell-to-cell shifts of boundaries, demarcating neighboring TADs.

- Chromatin domain clusters (CDCs) carry more compact and mostly repressed CDs in their interior, and less compact, transcriptionally competent CDs at their periphery. The latter are closely associated with IC-channels and wider lacunas, carrying splicing speckles and other nuclear bodies. This reasoning is consistent with a nanoscale zonation of euchromatic and heterochromatic regions in CDCs.
- CDs, which mostly contain transcriptionally competent or active chromatin, may include short segments with repressive epigenetic signatures, whereas a CD comprising mostly repressed chromatin, may carry short segments with epigenetic marks signifying their transcriptional competence. These segments (named compartmental domains[27]) may comprise only a few kb, even only a single transcription unit. An active gene, located within a repressed CD, provides an anomaly like a grain of sugar in a pepper box. Vice versa, a repressed gene, located within a mostly active CD, reminds of a grain of pepper in a sugar box. Such examples do not invalidate the current concept of a hierarchically defined structural and functional higher order chromatin organization but necessitate a reconsideration of this concept. More detailed comparisons between the nuclear landscapes present in different cell types and species are required to solve this problem.
- A role of IC-channels as structural and functional boundaries between CDs and CDCs located on both sides has been considered but neither experimentally proven nor falsified.

Manipulations of defined target DNA sequences and proteins, respectively, will help to test our present and other hypotheses as rigorously as possible, and to explore the molecular mechanisms involved in the impact of the formation, preservation and changes of structural entities on nuclear functions.

Supplementary Table 2: list of used antibodies

Antibodies	*Dilution*	*Supplier*	*Catalog number*
RAD21	1:200	Abcam	ab154769
SMC1	1:200	Bethyl laboratories	A300-055A
SMC3	1:200	Bethyl laboratories	A300-060A
Goat anti rabbit Cy3	1:200	Dianova	111-165-045
Mouse anti SC35	1:1000	Sigma	S4045
Mouse anti RNA Pol II Ser5P	1:500	Abcam	ab5408
Rabbit anti H3K27me3	1:500	Active Motif	39155
Donkey anti mouse Alexa 488	1:400	Life technologies	A21202
Donkey anti rabbit Alexa 594	1:400	Life technologies	A21207
Mouse anti digoxigenin Cy5	1:100	Sigma	D8156
Streptavidin Alexa 488	1:200	Invitrogen	S-11223

Supplementary References

1 Wojcik, C. & DeMartino, G. N. Intracellular localization of proteasomes. *Int J Biochem Cell Biol* **35**, 579-589, doi:10.1016/s1357-2725(02)00380-1 (2003).

2 Natsume, T., Kiyomitsu, T., Saga, Y. & Kanemaki, M. T. Rapid Protein Depletion in Human Cells by Auxin-Inducible Degron Tagging with Short Homology Donors. *Cell Rep* **15**, 210-218, doi:10.1016/j.celrep.2016.03.001 (2016).

3 Fei, J. *et al.* Quantitative analysis of multilayer organization of proteins and RNA in nuclear speckles at super resolution. *J Cell Sci* **130**, 4180-4192, doi:10.1242/jcs.206854 (2017).

4 Roukos, V., Pegoraro, G., Voss, T. C. & Misteli, T. Cell cycle staging of individual cells by fluorescence microscopy. *Nat Protoc* **10**, 334-348, doi:10.1038/nprot.2015.016 (2015).

5 Schermelleh, L., Heintzmann, R. & Leonhardt, H. A guide to super-resolution fluorescence microscopy. *J Cell Biol* **190**, 165-175, doi:jcb.201002018 [pii]10.1083/jcb.201002018 (2010).

6 Otterstrom, J. *et al.* Super-resolution microscopy reveals how histone tail acetylation affects DNA compaction within nucleosomes in vivo. *Nucleic Acids Res*, doi:10.1093/nar/gkz593 (2019).

7 Ricci, M. A., Manzo, C., Garcia-Parajo, M. F., Lakadamyali, M. & Cosma, M. P. Chromatin fibers are formed by heterogeneous groups of nucleosomes in vivo. *Cell* **160**, 1145-1158, doi:10.1016/j.cell.2015.01.054 (2015).

8 Boettiger, A. & Murphy, S. Advances in Chromatin Imaging at Kilobase-Scale Resolution. *Trends Genet* **36**, 273-287, doi:10.1016/j.tig.2019.12.010 (2020).

9 Cremer, T. *et al.* The 4D nucleome: Evidence for a dynamic nuclear landscape based on co-aligned active and inactive nuclear compartments. *FEBS Lett* **589**, 2931-2943, doi:10.1016/j.febslet.2015.05.037 (2015).

10 Cremer, T. *et al.* Chromosome territories, interchromatin domain compartment, and nuclear matrix: an integrated view of the functional nuclear architecture. *Crit Rev Eukaryot Gene Expr* **10**, 179-212 (2000).

11 Jackson, D. A. & Pombo, A. Replicon clusters are stable units of chromosome structure: evidence that nuclear organization contributes to the efficient activation and propagation of S phase in human cells. *J Cell Biol* **140**, 1285-1295, doi:10.1083/jcb.140.6.1285 (1998).

12 Ma, H. *et al.* Spatial and temporal dynamics of DNA replication sites in mammalian cells. *J Cell Biol* **143**, 1415-1425 (1998).

13 Miron, E. *et al.* Chromatin arranges in chains of mesoscale domains with nanoscale functional topography independent of cohesin. *Science Advances* **6**, eaba8811, doi:DOI: 10.1126/sciadv.aba8811 (2020).

14 Bintu, B. *et al.* Super-resolution chromatin tracing reveals domains and cooperative interactions in single cells. *Science* **362**, doi:10.1126/science.aau1783 (2018).

15 Mateo, L. J. *et al.* Visualizing DNA folding and RNA in embryos at single-cell resolution. *Nature* **568**, 49-54, doi:10.1038/s41586-019-1035-4 (2019).

16 Baddeley, D. *et al.* Measurement of replication structures at the nanometer scale using super-resolution light microscopy. *Nucleic Acids Res* **38**, e8, doi:10.1093/nar/gkp901 (2010).

17 Nakamura, H., Morita, T. & Sato, C. Structural organizations of replicon domains during DNA synthetic phase in the mammalian nucleus. *Exp Cell Res* **165**, 291-297, doi:10.1016/0014-4827(86)90583-5 (1986).

18 Xiang, W. *et al.* Correlative live and super-resolution imaging reveals the dynamic structure of replication domains. *J Cell Biol* **217**, 1973-1984, doi:10.1083/jcb.201709074 (2018).

19 Schmid, V. J., Cremer, M. & Cremer, T. Quantitative analyses of the 3D nuclear landscape recorded with super-resolved fluorescence microscopy. *Methods* **123**, 33-46, doi:10.1016/j.ymeth.2017.03.013 (2017).

20 Cremer, T. & Cremer, M. Chromosome territories. *Cold Spring Harb Perspect Biol* **2**, a003889, doi:10.1101/cshperspect.a003889 (2010).

21 Cremer, T. *et al.* The Interchromatin Compartment Participates in the Structural and Functional Organization of the Cell Nucleus. *Bioessays* **42**, e1900132, doi:10.1002/bies.201900132 (2020).

22 Fudenberg, G. *et al.* Formation of Chromosomal Domains by Loop Extrusion. *Cell Rep* **15**, 2038-2049, doi:10.1016/j.celrep.2016.04.085 (2016).

23 Lieberman-Aiden, E. *et al.* Comprehensive mapping of long-range interactions reveals folding principles of the human genome. *Science* **326**, 289-293, doi:10.1126/science.1181369 (2009).

24 Sanborn, A. L. *et al.* Chromatin extrusion explains key features of loop and domain formation in wild-type and engineered genomes. *Proc Natl Acad Sci U S A* **112**, E6456-6465, doi:10.1073/pnas.1518552112 (2015).

25 Rao, S. S. *et al.* A 3D map of the human genome at kilobase resolution reveals principles of chromatin looping. *Cell* **159**, 1665-1680, doi:10.1016/j.cell.2014.11.021 (2014).

26 Rao, S. S. P. *et al.* Cohesin Loss Eliminates All Loop Domains. *Cell* **171**, 305-320 e324, doi:10.1016/j.cell.2017.09.026 (2017).

27 Rowley, M. J. & Corces, V. G. Organizational principles of 3D genome architecture. *Nat Rev Genet* **19**, 789-800, doi:10.1038/s41576-018-0060-8 (2018).

28 Dixon, J. R., Gorkin, D. U. & Ren, B. Chromatin Domains: The Unit of Chromosome Organization. *Mol Cell* **62**, 668-680, doi:10.1016/j.molcel.2016.05.018 (2016).

29 Dixon, J. R. *et al.* Topological domains in mammalian genomes identified by analysis of chromatin interactions. *Nature* **485**, 376-380, doi:10.1038/nature11082 (2012).

30 Sexton, T. *et al.* Three-dimensional folding and functional organization principles of the Drosophila genome. *Cell* **148**, 458-472, doi:10.1016/j.cell.2012.01.010 (2012).

31 Szabo, Q. *et al.* TADs are 3D structural units of higher-order chromosome organization in Drosophila. *Sci Adv* **4**, eaar8082, doi:10.1126/sciadv.aar8082 (2018).

32 Marchal, C., Sima, J. & Gilbert, D. M. Control of DNA replication timing in the 3D genome. *Nat Rev Mol Cell Biol*, doi:10.1038/s41580-019-0162-y (2019).
33 Miura, H. *et al.* Single-cell DNA replication profiling identifies spatiotemporal developmental dynamics of chromosome organization. *Nature Genetics* **51**, 1356–1368 (2019).
34 Moindrot, B. *et al.* 3D chromatin conformation correlates with replication timing and is conserved in resting cells. *Nucleic Acids Res* **40**, 9470-9481, doi:10.1093/nar/gks736 (2012).
35 Pope, B. D. *et al.* Topologically associating domains are stable units of replication-timing regulation. *Nature* **515**, 402-405, doi:10.1038/nature13986 (2014).
36 Sima, J. *et al.* Identifying cis Elements for Spatiotemporal Control of Mammalian DNA Replication. *Cell* **176**, 816-830 e818, doi:10.1016/j.cell.2018.11.036 (2019).
37 Takahashi, S. *et al.* Genome-wide stability of the DNA replication program in single mammalian cells. *Nature Genetics* **51**, 529-540 (2019).
38 Zhao, P. A., Rivera-Mulia, J. C. & Gilbert, D. M. Replication Domains: Genome Compartmentalization into Functional Replication Units. *Adv Exp Med Biol* **1042**, 229-257, doi:10.1007/978-981-10-6955-0_11 (2017).
39 Beagrie, R. A. *et al.* Complex multi-enhancer contacts captured by genome architecture mapping. *Nature* **543**, 519-524, doi:10.1038/nature21411 (2017).
40 Cardozo Gizzi, A. M., Cattoni, D. I. & Nollmann, M. TADs or no TADS: Lessons From Single-cell Imaging of Chromosome Architecture. *J Mol Biol* **432**, 682-693, doi:10.1016/j.jmb.2019.12.034 (2020).
41 Shaban, H. A., Barth, R., Recoules, L. & Bystricky, K. Hi-D: Nanoscale mapping of nuclear dynamics in single living cells. *bioRxiv566638*, doi:doi: https://doi.org/10.1101/405969 (2020).
42 Waldeyer, W. Karyokinesis and its relation to the process of fertilization. *QJ microsc Sci* **30**, 159-281 (1888).
43 Cremer, T. & Cremer, C. Rise, fall and resurrection of chromosome territories: a historical perspective. Part I. The rise of chromosome territories. *Eur J Histochem* **50**, 161-176 (2006).
44 Cremer, T. & Cremer, C. Centennial of Wilhelm Waldeyer's introduction of the term "chromosome" in 1888. *Cytogenet. Cell Genet.* **48**, 65-67 (1988).
