# Supplementary material for: Cohesin depleted cells rebuild functional nuclear compartments after endomitosis
Source: Nat Commun. 2020 Dec 1;11:6146. doi: 10.1038/s41467-020-19876-6 (PMC7708632; doi:10.1038/s41467-020-19876-6)
Supplement: Supplementary file 3 — Description of Additional Supplementary Files [file 41467_2020_19876_MOESM3_ESM.pdf]

Description of Additional Supplementary Files

Supplementary Data 1: Time lapse imaging data of individual nuclei followed through mitosis

Left panel: control cells (n= 45); right panel: auxin treated cells (n=36) followed in parallel by live cell observation for 21h ($\Delta t_{\text{observation points}}$ =15min) to compare entrance into mitosis, mitotic progression and exit.

Nuclei highlighted in green: inconspicuous mitosis with formation of two daughter cells completed within 60 min in control nuclei (n=35; 77.8%) and in auxin treated nuclei (n=4; 11.1%). In auxin treated cells these mitoses were either recorded immediately after addition of auxin (time frame 1, compare e.g. cells 1 and 7) or in cells that escaped RAD21-mClover degradation (compare e.g. cells 34 and 35). Nuclei highlighted in gold: prolonged mitosis (>90 min) with formation of two daughter cells in control nuclei (n=1; 2.2%), not observed in auxin treated nuclei. Nuclei highlighted in light orange: prolonged mitosis with formation of n MLN in control nuclei (n=5; 11.1%) and in auxin treated nuclei (n=23; 63.9%). Nuclei highlighted in dark orange: prolonged mitosis (> 90min) in control nuclei (n=4; 8.9%) and in auxin treated nuclei (n=9; 25.0%), where mitotic exit could not be recorded within the observation period. Since all prolonged mitoses in cohesin depleted cells recorded over the entire mitosis resulted in one MLN, the same outcome of these mitoses can be assumed.

Supplementary Software: Custom source code for Python and R used for quantification of RAD21-mAID-mClover degradation and DAPI intensity measurement

The custom R script “Data_Normalization.R” was used to process median intensity data of single cells extracted from Fiji to subtract background. The custom Python script “Auxin_treatment_timeseries.ipynb” was used to process normalized (background subtracted) median intensity data extracted from images of single live cells tracked over time and generate plots for quantitation of auxin induced RAD21-mAID-mClover degradation in single live cells in Supplementary Fig. 2b. The custom R script “DAPI_content.R” was used to integrate all DAPI intensity measurements for DNA content analysis after normalization and generate the plot of DNA content in individual nuclei shown in Supplementary Fig. 5. Software is provided as one .zip file.
