# Supplementary material for: Cohesin depleted cells rebuild functional nuclear compartments after endomitosis
Source: Nat Commun. 2020 Dec 1;11:6146. doi: 10.1038/s41467-020-19876-6 (PMC7708632; doi:10.1038/s41467-020-19876-6)
Supplement: Supplementary file 6 — Reporting Summary [file 41467_2020_19876_MOESM6_ESM.pdf]

natureresearch

Corresponding author(s): Thomas Cremer, Marion Cremer, Hartmann Harz

Last updated by author(s): Oct 14, 2020

Reporting Summary

Nature Research wishes to improve the reproducibility of the work that we publish. This form provides structure for consistency and transparency in reporting. For further information on Nature Research policies, see Authors & Referees and the Editorial Policy Checklist .

Statistics

For all statistical analyses, confirm that the following items are present in the figure legend, table legend, main text, or Methods section.

n/a	Confirmed	
☐	☒	The exact sample size (*n*) for each experimental group/condition, given as a discrete number and unit of measurement
☐	☒	A statement on whether measurements were taken from distinct samples or whether the same sample was measured repeatedly
☐	☒	The statistical test(s) used AND whether they are one- or two-sided<br>*Only common tests should be described solely by name; describe more complex techniques in the Methods section.*
☐	☒	A description of all covariates tested
☒	☐	A description of any assumptions or corrections, such as tests of normality and adjustment for multiple comparisons
☐	☒	A full description of the statistical parameters including central tendency (e.g. means) or other basic estimates (e.g. regression coefficient) AND variation (e.g. standard deviation) or associated estimates of uncertainty (e.g. confidence intervals)
☐	☒	For null hypothesis testing, the test statistic (e.g. *F*, *t*, *r*) with confidence intervals, effect sizes, degrees of freedom and *P* value noted<br>*Give P values as exact values whenever suitable.*
☒	☐	For Bayesian analysis, information on the choice of priors and Markov chain Monte Carlo settings
☒	☐	For hierarchical and complex designs, identification of the appropriate level for tests and full reporting of outcomes
☒	☐	Estimates of effect sizes (e.g. Cohen's *d*, Pearson's *r*), indicating how they were calculated

Our web collection on statistics for biologists contains articles on many of the points above.

Software and code

Policy information about availability of computer code

Data collection	Confocal fluorescence microscopy: Leica Application Suite X (version 3.5.2.18963), Leica<br>Spinning disk confocal microscopy: NIS Elements (version 5.02.00), Nikon<br>Structured illumination microscopy: DeltaVisionOMX (version 2.25), SoftWoRx (version 5.1.0), Applied Precision Imaging/GE Healthcare<br>Operetta: Harmony (version 3.5.1), Perkin Elmer<br>Widefield microscopy: Axiovision AxioVs 40V (version 4.8.2.0 SP3), Zeiss
Data analysis	The software employed for DAPI intensity classification (Fig. 3 and 4) is implemented in the R package nucim and the accompanying package bioimagetools. Additional information and development versions of both packages can be found on https://bioimaginggroup.github.io/nucim and https://bioimaginggroup.github.io/bioimagetools. Detailed description is found in Schmid et al. (2017). Quantitative allocation of defined nuclear targets on 3D chromatin compaction classes were performed using custom built scripts for the open-source statistical software R http://www.r-project.org, available on request). Signal segmentation and quantification for object counting and volume (Fig. 8) is based on commercially available volocity software and performance described in the Methods section in detail. Image analysis for quantification of RAD21 decay of live cell timelapses (Suppl Fig. 2) was performed using Ilastik (standard settings), Fiji and custom Python and R scripts available under https://github.com/CALM-LMU/Cohesin_project.git. A brief description can be found in the Methods section. The detailed description of segmentation and analysis scripts can be found as comments in the scripts deposited on GitHub. For DNA content analysis (Suppl Fig. 5) Ilastik (standard settings), Fiji and custom R scripts (https://github.com/CALM-LMU/Cohesin_project.git) were used. The analysis is briefly described in the Methods section and in detail as comments in the scripts deposited on GitHub.<br>All Hi-C data was processed using the software package Juicer version 1.5.7, which can be found at https://github.com/aidenlab/juicer. Previously published ChIP-Seq data from Rao et al. Cell 2017 was clustered using the scipy.cluster.vq.kmeans function. Repli-Seq data was processed and analyzed exactly following the code published in Marchal et al., Nature Protocols 2018.

For manuscripts utilizing custom algorithms or software that are central to the research but not yet described in published literature, software must be made available to editors/reviewers. We strongly encourage code deposition in a community repository (e.g. GitHub). See the Nature Research guidelines for submitting code & software for further information.

Data

Policy information about availability of data

All manuscripts must include a data availability statement. This statement should provide the following information, where applicable:

- Accession codes, unique identifiers, or web links for publicly available datasets
- A list of figures that have associated raw data
- A description of any restrictions on data availability

All HiC and Repli-Seq data (processed and raw) generated as part of this study (fig. 5 and 7) can be found under https://www.ncbi.nlm.nih.gov/geo/query/acc.cgi?acc=GSE145099 with GEO accession: GSE145099. Publicly available ChIP-Seq data used in this study are available at GEO accession: GSE104888 (https://www.ncbi.nlm.nih.gov/geo/query/acc.cgi?acc=GSE104888).
Raw data used for Figs. 1-4, 6, 8, Supplementary Figs. 1-3 and 5-8, additional „biological replicates" and complementary experiments can be accessed under https://doi.org/10.5061/dryad.vt4b8gtqb.

Field-specific reporting

Please select the one below that is the best fit for your research. If you are not sure, read the appropriate sections before making your selection.

☒ Life sciences ☐ Behavioural & social sciences ☐ Ecological, evolutionary & environmental sciences

For a reference copy of the document with all sections, see nature.com/documents/nr-reporting-summary-flat.pdf

Life sciences study design

All studies must disclose on these points even when the disclosure is negative.

Sample size

A predetermined sample size calculation was not performed. Variable sample sizes were chosen depending on the addressed questions and microscopic/evaluation approaches.
Fig. 1 and Supplementary data file 1: live cell analyses were based on a total of 29 and 32 randomly chosen observation areas monitored in parallel for 20h for controls and auxin treated cells, respectively. From these data sets 45 and 36 cells respectively could be extracted for a full 20h monitoring (Supplementary data file 1). Statistical analysis was not performed.
Fig. 2: A minimum of 20 nuclei /series were recorded. No statistical analysis was performed.
Fig. 3, 4 (with Suppl. Fig. 7): Between 16-39 nuclei/series were used for quantitative evaluations from 3D-SIM recorded image stacks of entire nuclei. These data stacks provided several thousand individual fluorescence signals/nucleus and were used for statistical analysis.
Fig. 8: Between 7-11 nuclei/series were used for quantitative evaluations from 3D-SIM recorded image stacks of entire nuclei. These data stacks provided several thousand individual fluorescence signals/nucleus and were used for statistical analysis.
Suppl Fig. 1B: A total of >20.000 evaluated nuclei ensured a representative dataset for statistical evaluation.
Suppl. Fig. 2: For the requested observation period over 6h a total of n=82 (control) and n=69 (auxin treated) randomly selected cells were chosen. Statistical evaluation was not performed.
Suppl. Fig. 3: ~1000 cells/series were evaluated to ensure a representative dataset based on randomly selected microscopic observation fields with all nuclei implemented in the evaluation. Statistical evaluation was not performed.
Suppl. Fig. 5: 19 control cells and 20 postmitotic cohesin depleted MLN after 30h auxin treatment from randomly acquired image fields were used to measure DNA contents. No statistical analysis was performed.
Suppl. Fig. 8: A total of n=56 (control) and 80 (auxin treated) nuclei were evaluated. These numbers reflect the total number of nuclei found for each sample in the four biological replicates and were used for statistical analysis.

For E/L Repli-seq 200k cells were sorted from each S phase fraction. This sample size is considered sufficient for Repli-seq.
For Hi-C, ~3M cells were fixed with formaldehyde crosslinking cells for each timepoint. At least two replicates for each time point were performed in line with the ENCODE consortium experimental standards.

Data exclusions

For Repli-Seq analysis sex chromosomes are excluded from analysis because of the large differences in allelic replication timing between the active and inactive X chromosome.

Replication

Experiments for microscopic studies were performed at least two times for each condition and confirmed reproducibility of results. Each replication was successful. For Hi-C at least two replicates of each timepoint were performed in line with the ENCODE consortium experimental standards. All attempts at replication were successful. All findings described in the manuscript were confirmed in individual replicates.

Randomization

Randomization in our experiments is the process of allocating experimental units via random assignment to auxin treatment or control condition. A cell culture with tens of thousands of cells can be assumed to be randomized (e.g. for cell cycle state). In our case, control and auxin-treated cells used for an experiment were derived from a common cell culture that was split into identical cell culture devices. One of them was treated with auxin, the other was used as a control. There was no preselection (e.g. based on cell density) of these sub cultures. Randomization was not necessary as we did not allocate datasets into experimental groups.

Blinding

The effects seen in auxin treated cultures with regard to differences in mitotic index and abnormal nuclear morphologies are so evident and obvious that a blinding did not make sense.

Reporting for specific materials, systems and methods

We require information from authors about some types of materials, experimental systems and methods used in many studies. Here, indicate whether each material, system or method listed is relevant to your study. If you are not sure if a list item applies to your research, read the appropriate section before selecting a response.

Materials & experimental systems

n/a	Involved in the study
☐	☒ Antibodies
☐	☒ Eukaryotic cell lines
☒	☐ Palaeontology
☒	☐ Animals and other organisms
☒	☐ Human research participants
☒	☐ Clinical data

Methods

n/a	Involved in the study
☒	☐ ChIP-seq
☒	☐ Flow cytometry
☒	☐ MRI-based neuroimaging

Antibodies

Antibodies used

Antibodies Source Catalog number
RAD21 Abcam ab154769
SMC1 Bethyl laboratories A300-055A
SMC3 Bethyl laboratories A300-060A
Mouse anti SC35 Sigma S4045
Mouse anti RNA Pol II Ser5P Abcam ab5408
Rabbit anti H3K27me3 Active Motif 39155
Donkey anti mouse Alexa 488 Life technologies A21202
Donkey anti rabbit Alexa 594 Life technologies A21207
Cy3-conjugated goat anti rabbit Dianova 111-165-045
Cy5-conjugated mouse-anti-DIG Sigma D8156
Streptavidin-Alexa-488 Invitrogen S-11223

Validation

RAD21 Abcam ab154769: for validation see https://www.abcam.com/rad21-antibody-ab154769.html
SMC1 Bethyl laboratories A300-055A: for validation see https://www.bethyl.com/product/A300-055A/SMC1+Antibody
SMC3 Bethyl laboratories A300-060A: for validation see https://www.bethyl.com/product/A300-060A/SMC3+Antibody
Mouse anti SC35 Sigma S4045: for validation see https://www.sigmaaldrich.com/catalog/product/sigma/s4045
Mouse anti RNA Pol II Ser5P Abcam ab5408: for validation seehttps://www.abcam.com/rna-polymerase-ii-ctd-repeat-ysptsps-phospho-s5-antibody-4h8-chip-grade-ab5408.html
Rabbit anti H3K27me3 Active Motif 39155: for validation see https://www.activemotif.com/catalog/details/39155
Cy3-conjugated goat anti rabbit Dianova 111-165-045: for validation see https://www.dianova.com/downloads/Jackson/111-165-045.pdf
Cy5-conjugated mouse-anti-DIG Sigma D8156: for validation see https://www.sigmaaldrich.com/catalog/product/sigma/d8156?lang=de&region=DE
Streptavidin-Alexa-488 Invitrogen S-11223: for validation see https://www.thermofisher.com/order/catalog/product/S11223#/S11223

Eukaryotic cell lines

Policy information about cell lines

Cell line source(s)

The transgenic cell line HCT11-RAD21-mAID-mClover cell was established in the lab of Masato Kanemaki by Toyoaki Natsume (Center of Frontier Research, National Institute of Genetics, Mishima, Shizuoka Japan). For consistency aliquots of these cells previously used in the work of Rao et al., Cell, (2017) were used.

Authentication

Efficient Auxin-inducible RAD21 degradation was confirmed by loss of mClover fluorescence coupled to RAD21 upon auxin addition (Suppl Fig. 1, Suppl Fig. 2). The cell line was authenticated by its expected loss of mClover fluorescence coupled to RAD21 and the specific loss of RAD21 immunodetection upon addition of auxin.

Mycoplasma contamination

The used cell line was tested for mycoplasm contamination by microscopic inspection after DAPI staining . Cells were re-evaluated after each thawing of a new vial. All used samples were found negative for mycoplasms.

Commonly misidentified lines
(See ICLAC register)

none
